# Role of Interphase in the Mechanical Behavior of Silica/Epoxy Resin Nanocomposites

**DOI:** 10.3390/ma8063519

**Published:** 2015-06-16

**Authors:** Yi Hua, Linxia Gu, Sundaralingam Premaraj, Xiaodong Zhang

**Affiliations:** 1 Department of Mechanical and Materials Engineering, University of Nebraska-Lincoln, Lincoln, NE 68588-0656, USA; E-Mail: huayixiaolu@gmail.com; 2 Nebraska Center for Materials and Nanoscience, Lincoln, University of Nebraska-Lincoln, NE 68588-0656, USA; E-Mail: lgu@unl.edu; 3 Department of Growth & Development, University of Nebraska Medical Center, College of Dentistry, Lincoln, NE 68583-0750, USA; E-Mail: spremaraj@unmc.edu; 4 Key Laboratory of Education Ministry for Modern Design and Rotor-Bearing System, Xi’an Jiaotong University, Xi’an 710049, China

**Keywords:** nano-structures, interface/interphase, mechanical properties, computational modeling, representative volume element

## Abstract

A nanoscale representative volume element has been developed to investigate the effect of interphase geometry and property on the mechanical behavior of silica/epoxy resin nanocomposites. The role of interphase–matrix bonding was also examined. Results suggested that interphase modulus and interfacial bonding conditions had significant influence on the effective stiffness of nanocomposites, while its sensitivities with respect to both the thickness and the gradient property of the interphase was minimal. The stiffer interphase demonstrated a higher load-sharing capacity, which also increased the stress distribution uniformity within the resin nanocomposites. Under the condition of imperfect interfacial bonding, the effective stiffness of nanocomposites was much lower, which was in good agreement with the documented experimental observations. This work could shed some light on the design and manufacturing of resin nanocomposites.

## 1. Introduction

Resin-based composites possess good aesthetic properties and are currently among the most popular dental restorative materials [1]. However, their wear resistance, hardness, and shrinkage behaviors are still a concern. Nanoparticle-reinforcement has become an effective technique to improve the mechanical performance of these composites [2,3]. Due to the large surface-to-volume ratio of the nanoparticles as well as the use of nanoparticle coatings, the molecular structure of the resin matrix is altered at the nanoparticle/matrix interface and the amount of this perturbed zone, referred to as interphase, could be substantial [4,5]. Although it is widely accepted that the interphase can significantly affect the performance of nanocomposites, the reported interphase property and thickness vary from case to case [6,7,8]. Yu *et al.* [9] estimated the properties of Al_2_O_3_ nanoparticle-reinforced epoxy composites through molecular dynamic simulations. They reported that the interphase was stiffer than the matrix, and its thickness was much smaller than the radius of the nanoparticles. To the contrary, Odegard *et al.* [10] studied the silica nanoparticle-reinforced polyimide composites using the same method and reported an interphase zone softer than the matrix while its thickness was comparable with the radius of the nanoparticles. In addition, the influence of interphase on the bonding strength between the nanoparticle and the matrix is also not clear. Lauke [11] analyzed the stress state around a coated particle in a polymer matrix to determine the adhesion strength at the particle–matrix interface. Boutaleb *et al.* [12] developed a micromechanical analytical model to predict the yield stress of nanocomposites accounting for an interphase around the nanoparticles and found out that this zone played a key role on the yield stress of nanocomposites. Zappalorto *et al.* [13] developed a closed form expression for the critical debonding stress and showed that the interphase properties, linked to surface functionalizers, significantly affected the debonding stress, especially for nanoparticle radii below 50 nm. Zhang *et al.* [14] have experimentally demonstrated that the bonding strength at the interphase–matrix interface was weaker than that at the interphase-nanoparticle interface. However, existing approaches generally assumed perfect bonding between the interphase and the matrix [15,16], while the case of imperfect bonding is lacking [17,18], especially for nanoparticle-reinforced composites.

The aim of this paper is to investigate the role of interphase in the mechanical behavior of silica/epoxy resin nanocomposites by developing a nanoscale representative volume element (RVE). The effect of imperfect bonding between the interphase and the matrix has also been considered. Results are expected to provide insights on the variation rule of the interphase towards optimizing the performance of silica/epoxy resin nanocomposites.

## 2. Finite Element Modeling

**Figure 1:**
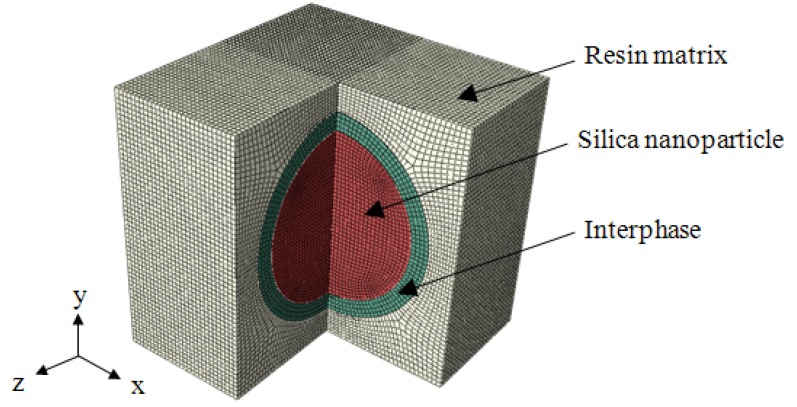
Three-dimensional representative volume element in a one-quarter view.

The configuration of the silica/epoxy resin nanocomposites was represented by a three-dimensional RVE with a length of 30 nm each side, as shown in Figure 1. This model consisted of three phases: a spherical silica nanoparticle with an annular interphase embedded inside the resin matrix. The diameter of the nanoparticle varied from 14 to 25 nm, leading to a range of nanoparticle volume fraction from 5% to 30%. A uniform thickness of 2 nm was initially assumed for the interphase. The material properties of the epoxy resin matrix were Young’s modulus *E*_m_ = 1.7 GPa and Poisson’s ratio ν_m_ = 0.4. For silica nanoparticle, they were *E*_p_ = 22 GPa and ν_p_ = 0.38 [19]. The interphase was initially assumed to have the same material properties as the matrix, *i.e.*, *E*_i_ = *E*_m_ and ν_i_ = ν_m_, due to the reported controversy in identifying these properties. Here the subscripts m was denoted as the matrix, i as the interphase, and p as the nanoparticle.

All three phases were meshed with reduced 8-node hexahedral elements (C3D8R) using a commercial finite element software ABAQUS (Dassault Systems Simulia Corp., RI, USA). A mesh convergence test was performed and the mesh size of 0.5 nm was selected. Uniaxial tension along the x-direction was implemented by a 3-nm displacement. Periodic boundary condition, which developed by a user-specified Python script, was enforced in all directions to extend the RVE periodically, *i.e.*, considering the interaction between the RVE with its mirrored images. The periodic boundary condition was expressed in terms of the displacement vector **u**, which related the displacements between the opposite edges according to

**u** (x, y, 0) − **u**_z_ = **u** (x, y, *L*)

**u** (x, 0, z) − **u**_y_ = **u** (x, *L*, z)

**u** (0, y, z) − **u**_x_ = **u** (*L*, y, z)

where *L* was the length of the RVE; x, y, and z stood for the coordinate axes of the three edges of the RVE; and **u**_x_, **u**_y_, and **u**_z_ depended on the particular loading applied to the RVE. Two bonding conditions, imperfect bonding, *i.e.*, tangential sliding with the friction coefficient of 0.1, and perfect bonding, *i.e.*, surface-based tie constraint, were considered at the interphase-matrix interface for comparison.

## 3. Results

### 3.1. Model Verification

**Figure 2:**
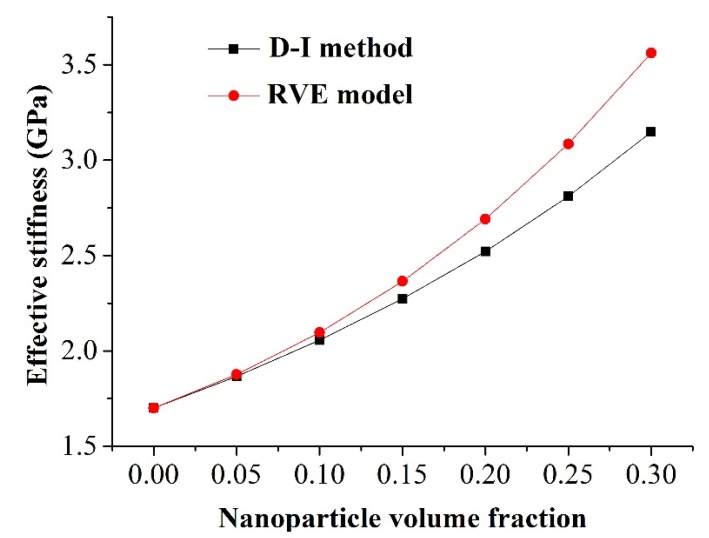
Comparison of the effective stiffness obtained by the representative volume element model and the double-inclusion method.

Our simulation results were compared with the exact solutions obtained from the classical double-inclusion (D-I) method proposed by Nemat-Nasser and Hori [20]. The comparison results are shown in Figure 2. It is observed that the effective stiffness predicted by the RVE model was higher than that obtained from the D-I method. For the nanoparticle volume fraction of 10%, the effective stiffness predicted by the RVE model was 1.9% higher than that calculated by the D-I method. As the nanoparticle volume fraction increased to 30%, the maximum deviation of the effective stiffness was 13.1% between the FE prediction and the theoretical solution. The discrepancies increase with higher nanoparticle volume fraction, which has also been reported by Lin *et al.* [21]. This could be explained by the simplifications of the D-I method, which does not consider the nanoparticle interaction effect.

### 3.2. Influence of Interphase Modulus and Thickness

**Figure 3:**
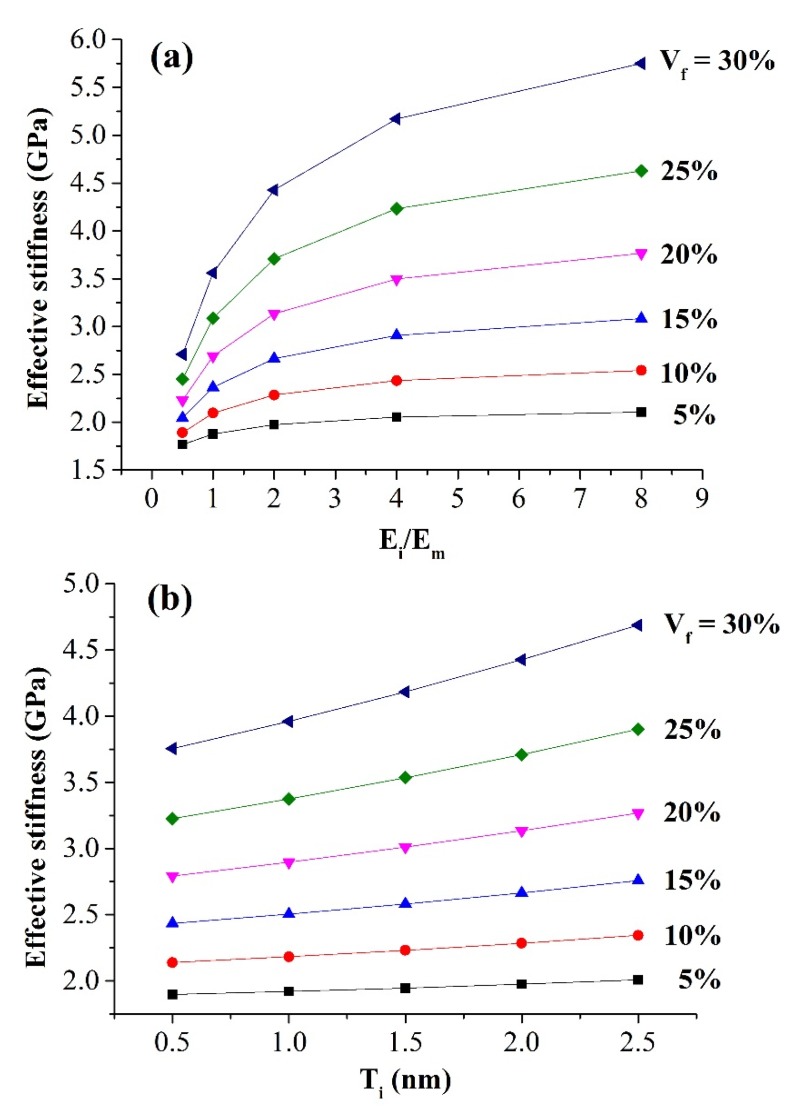
Effect of (**a**) interphase modulus and (**b**) interphase thickness on the effective stiffness of nanocomposites.

Relative softer and stiffer interphase materials were systematically investigated as shown in Figure 3a. The Young’s modulus of the interphase (*E*_i_) varied from 0.5 *E*_m_ as 0.85 GPa to 8 *E*_m_ as 13.6 GPa while the thickness of the interphase (*T*_i_) was kept constant at 2 nm. It is clear that the effective stiffness of nanocomposites increased with an increase in interphase modulus. The sensitivity of this effective stiffness with respect to the interphase modulus increased with a larger nanoparticle volume fraction. For example, there was a maximum 112% increase in the effective stiffness when the interphase modulus increased from 0.5 *E*_m_ to 8 *E*_m_ at the nanoparticle volume fraction of 30%. In addition, the growth rate of the effective stiffness decreased with larger interphase modulus. For a nanoparticle volume fraction of 20%, the growth rate of the effective stiffness per every 1.7 GPa was only 1.9% at the interphase modulus of 6.8 GPa (4 *E*_m_), compared to 41.4% at the interphase modulus of 0.85 GPa (0.5 *E*_m_).

The influence of interphase thickness on the mechanical behavior of nanocomposites is coupled to the interphase modulus. As the interphase modulus was larger than the matrix modulus, a thicker interphase led to stiffer nanocomposites, and *vice versa*. Figure 3b depicts the effective stiffness of nanocomposites by varying the interphase thickness (T_i_) from 0.5 to 2.5 nm and keeping the interphase modulus (*E*_i_) at 2 *E*_m_ of 3.4 GPa. Compared to the interphase modulus, the effective stiffness of nanocomposites was less sensitive to the interphase thickness, especially at lower nanoparticle volume fraction. For a nanoparticle volume fraction of 5%, the maximum increase in the effective stiffness was only 5.8%, compared to 24.8% at the 30% nanoparticle volume fraction.

### 3.3. Influence of Bonding Condition at the Interphase–Matrix Interface

**Figure 4:**
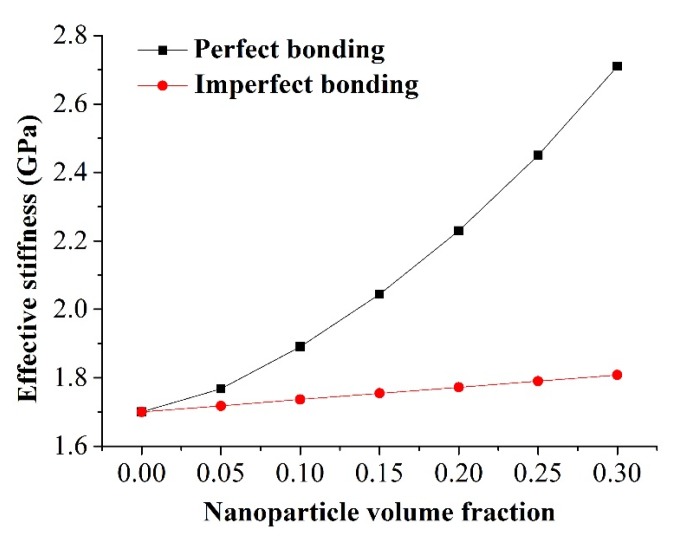
Effect of bonding condition between the interphase and the matrix on the effective stiffness of nanocomposites.

Two bonding conditions at the interphase-matrix interface were considered. One was with perfect bonding, and the other allowed tangential sliding with a friction coefficient of 0.1, hereby referred to as the imperfect bonding. The interphase property and geometry parameters were set as *E*_i_ = 0.85 GPa and T_i_ = 2 nm, respectively. The effect of both bonding conditions on the effective stiffness of nanocomposites is depicted in Figure 4. It is obvious that a perfect bonding assumption led to an overestimation of the effective stiffness of nanocomposites. This overestimation was exaggerated with an increase in the nanoparticle volume fraction. For a nanoparticle volume fraction of 10%, the effective stiffness under the perfect bonding at the interphase-matrix interface was 8.9% higher than that with the imperfect bonding, compared to 49.9% for a 30% nanoparticle volume fraction.

### 3.4. Gradient of Modulus in the Interphase

A constant gradient of 4 GPa with modulus varying from 6 to 18 GPa across the interphase was assumed as suggested by Lee *et al.* [22]. The model results were compared to a simplified model with a constant interphase modulus of 12 GPa, which was the average of the gradient interphase case. The predicted effective stiffness of nanocomposites was 3.007 GPa for the gradient case, and 3.057 GPa for the average case, respectively. The difference was less than 2%.

## 4. Discussion

Interphase properties of the nanoparticles varied due to the different treatment techniques, especially for those with a diameter less than 100 nm [13]. It is crucial to fully understand the influence of the interphase on the overall mechanical behavior of silica/epoxy resin nanocomposites. In this work, the effective stiffness of nanocomposites was investigated through the numerical model of a nanoscale RVE. The computational framework has been verified against the theoretical solution (Figure 2) as well as the published experimental data in our previous work [23]. The effects of the interphase modulus, thickness, as well as the bonding condition at the interphase–matrix interface were systematically characterized.

**Figure 5:**
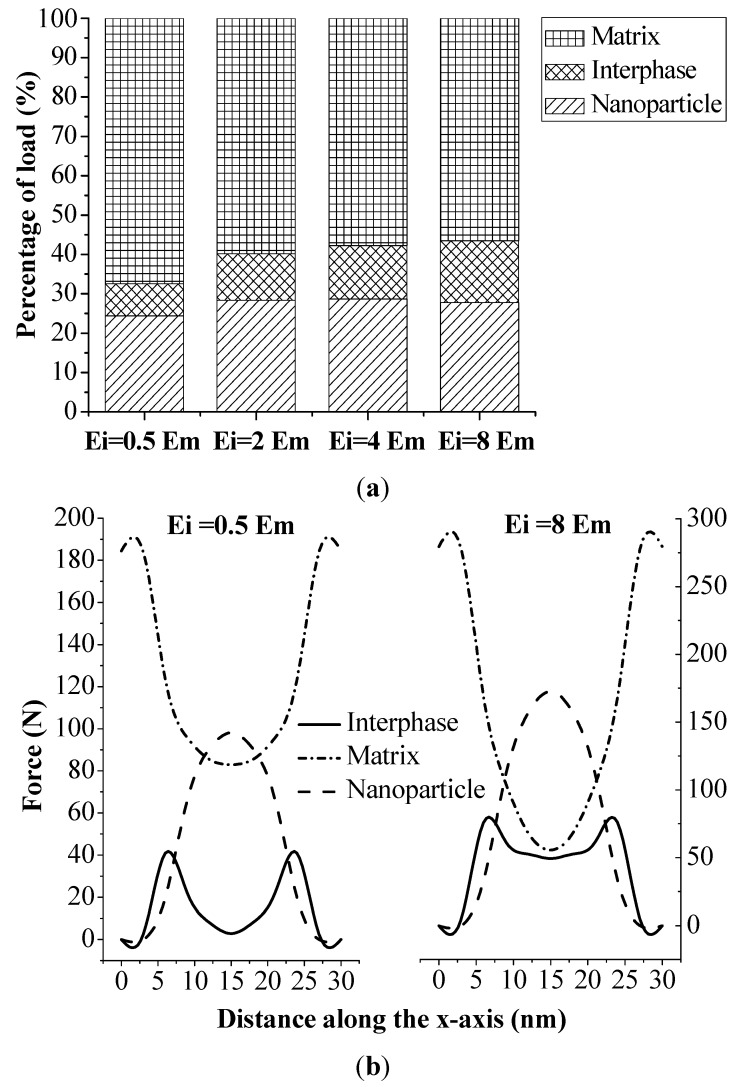
Effect of interphase modulus on (**a**) relative load-sharing capacity of the three-phase material; and (**b**) resultant forces of the three-phase material on the cross section along the loading direction.

Our results (Figure 3) clearly show that the interphase with a larger modulus significantly improved the effective stiffness of nanocomposites, while the stiffness sensitivity with respect to the interphase thickness was much less. The effect of the interphase modulus could be further explained by the load-sharing capacity of this three-phase material, as shown in Figure 5. Figure 5a depicts the relative load shared by each phase with the interphase modulus changing from 0.5 E_m_ to 8 E_m_ while the nanoparticle volume fraction was kept constant at 15%. The load shared by each phase was calculated as the integration of all nodal forces along the loading direction (x-axis). Results show that the load shared by the interphase and the nanoparticle increased 7.6% and 3.3%, respectively, when the interphase modulus varied from 0.5 *E*_m_ to 8 *E*_m_. In the meantime, the load shared by the matrix decreased 23.8%. The percentage kept as a plateau with further increasing the modulus of the interphase. This could be explained by the loading transfer between the matrix and the nanoparticle, *i.e.*, the maximum loading capacity of the material was limited by the volume fraction of stiffer phases. Figure 5b further depicts the resultant forces applied on each cross section, perpendicular to the loading direction, of each phase. The integration of each curve led to the actual load shared by each phase in Figure 5a. Three symmetric curves demonstrated the load variation of each phase along the loading direction. The matrix undertook almost the entire loading at both end surfaces, where no particle and interphase existed. The nanoparticle and the interphase started to share the loading as they appeared in the cross section. At the center of the RVE, the nanoparticle became the main load-bearing phase. Moreover, with the increase of the interphase modulus, the load shared by the interphase increased accordingly. This variation pattern indicates that the geometry and volume of the interphase are important factors for estimating the bulk behavior of nanocomposite material.

**Figure 6:**
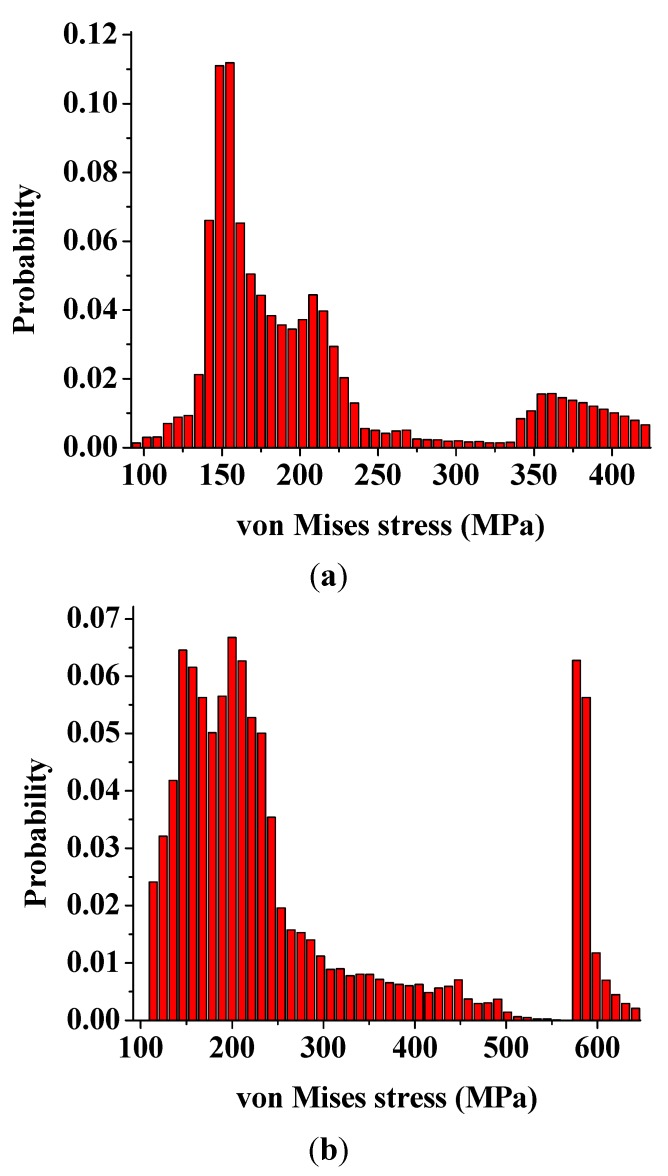
Probability distributions of the von Mises stress of nanocomposites for (**a**) *E*_i_ = 0.85 GPa; and (**b**) *E*_i_ = 3.4 GPa.

**Figure 7:**
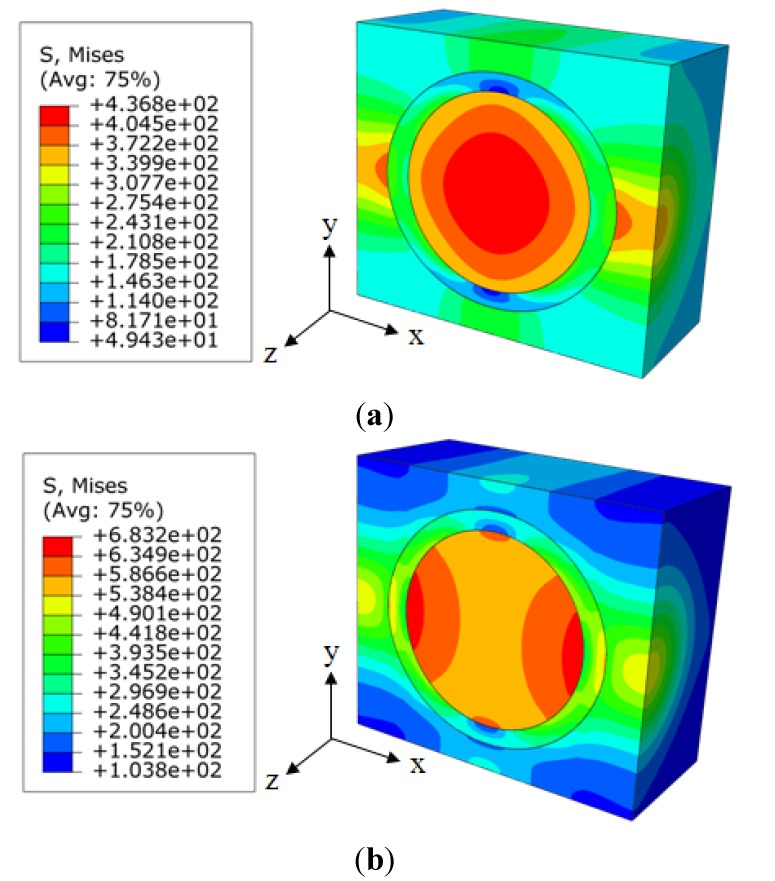
Von Mises stress distribution of nanocomposites for (**a**) *E*_i_ = 0.85 GPa; and (**b**) *E*_i_ = 3.4 GPa.

The increased load-sharing capacity of the interphase with a larger modulus also altered the peak stress components as well as the stress distributions within the nanocomposites. Figure 6 illustrates the probability distributions of the von Mises stress for the RVE models with the same nanoparticle volume fraction of 15% and different interphase modulus of 0.85 GPa (0.5 *E*_m_) and 3.4 GPa (2 *E*_m_), respectively. It is clear that the interphase with a larger modulus provided a higher peak stress and a relatively uniform stress distribution in the nanocomposites. In addition, both probability distributions have two clusters. The right cluster corresponded to the higher stress value, which was mainly located inside the nanoparticle and a small portion of it located in the other two phases near the central axis along the loading direction. While the left cluster, also the larger one, mainly related to the locations inside both relative softer interphase and matrix. The cluster stress distribution could be further visualized in Figure 7. It is observed that the stress concentration existed at both ends of the nanoparticle along the loading direction for the interphase with a larger modulus, corresponding to the right small peak in Figure 6b. This stress distribution also demonstrates a gradual transition from the low stress to the high stress inside the matrix, which corresponds to the smoother probability distribution of the left cluster in Figure 6b.

**Figure 8:**
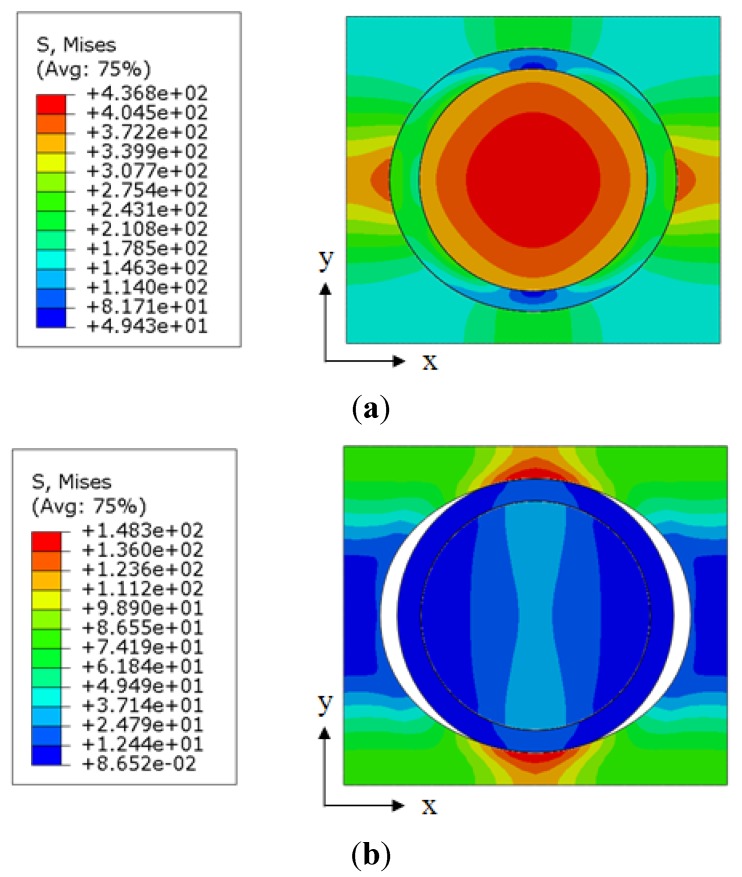
Stress distribution in the representative volume element models with different bonding conditions at the interphase–matrix interface: (**a**) perfect bonding; and (**b**) imperfect bonding (cut view in transverse plane).

Recent studies indicate that the interphase with smaller modulus might be the weakest link in the load path, and a potential debonding may occur in or near this region [24]. To examine this hypothesis, we built two RVE models that had the same interphase modulus of 0.85 GPa (0.5 *E*_m_) and different bonding conditions at the interphase–matrix interface. The stress distributions within the nanocomposites are shown in Figure 8. A gradient of the stress distribution from the matrix to the interphase and then to the nanoparticle was observed for the model with perfect bonding, as shown in Figure 8a. The maximum stress of 436.8 MPa was found to occur at the center of the nanoparticle. The stress distribution in the model, which assumed imperfect bonding between the interphase and the matrix, is shown in Figure 8b. A debonding was observed between the interphase and the matrix along the loading direction. As a result, most of the loads were carried by the matrix, rather than the nanoparticle, and the maximum stress of 148.3 MPa was located in the matrix normal to the loading direction. Our resulting debonding between the interphase and the matrix matches with the experimental observation by Zhang *et al.* [14].

**Figure 9:**
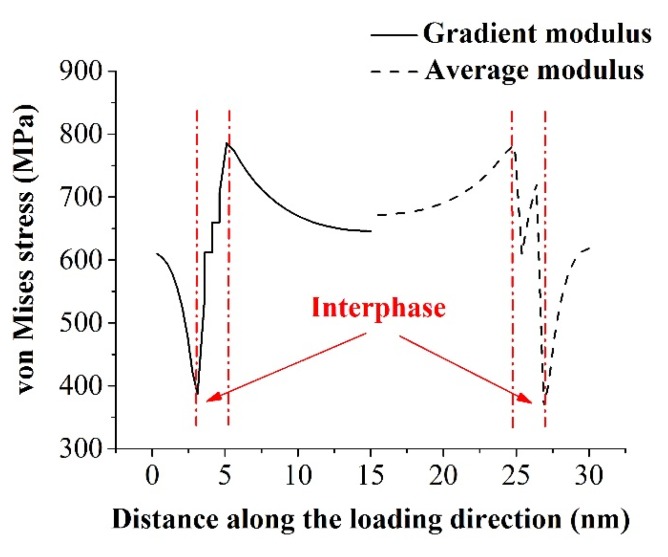
Von Mises stress distributions in the nanocomposites along the loading direction for the interphase with gradient modulus varied from 6 to 18 GPa or with the average modulus of 12 GPa.

Based on the nanoindentation test by Lee *et al.* [22], the elastic modulus in the interphase zone of a cellulose fiber-reinforced polypropylene composite varied across the interphase region. We found out that this gradient of modulus in the interphase had minimal impact on predicting the effective stiffness of nanocomposites. It should be noted though that the gradient of modulus in the interphase could alter the stress distributions in the nanocomposites, especially within the interphase region (Figure 9). Only half of the stress distribution along the loading path was depicted due to the symmetry of the RVE model. A relative smooth stress distribution was clearly demonstrated for the case with gradient modulus across the interphase. On the contrary, the stress concentration was observed for the case with average modulus in the interphase. This could be explained by the material mismatch between different phases of nanocomposites.

## 5. Conclusions

In this work, a nanoscale RVE was developed to investigate the effect of the interphase geometry and property on the mechanical performance of silica/epoxy resin nanocomposites. The effect of the interphase-matrix interaction on the bulk properties of nanocomposites was also considered. The accuracy of this model was verified by the solutions obtained from the classical D-I method. Our results have shown that an interphase with a larger modulus led to a higher load-sharing capacity, a reduced stress concentration at the interface, as well as a higher effective stiffness. In contrast, the influence of the interphase thickness on the effective stiffness of nanocomposites was minimal. We also demonstrated that the interphase with gradient property could be simplified as its average property without affecting the prediction on the bulk property of nanocomposites. Moreover, when imperfect bonding between the interphase and the matrix was considered, the effective stiffness decreased significantly. This could be attributed to the debonding that occurred between the interphase and the matrix, which has already been observed in previous published experimental work [14].

It should be noted that the Young’s modulus of silica nanoparticles ranges from 22 GPa, adopted in this work [19], to 70 GPa [25]. The calculated effective stiffness using a relative stiffer nanoparticle deviated from the presented results (Figure 2) up to 10.3% at the volume fraction of 30%. The obtained understanding on the role of interphase was unaltered considering the comparative nature of this work. In addition, the geometry of the RVE neglected the potential influence of nanoparticle distributions, such as clustering. A multiscale model considering both the nanoparticle network topology and the detailed nanoparticle interphase could be considered in the future. Despite all these simplifications, this study provides a fundamental understanding of the effect of the interphase on the bulk behavior of silica/epoxy resin nanocomposites, which could be used to guide the optimization of interphase parameters to achieve desirable mechanical properties of resin-based dental nanocomposites.
